# Supplementary material for: The Impact of Parenting Avoidance (IPA): Scale Development and Psychometric Evaluation Among Parents of Transgender Youth
Source: Behav Sci (Basel). 2025 May 3;15(5):625. doi: 10.3390/bs15050625 (PMC12109312; doi:10.3390/bs15050625)
Supplement: Supplementary file 1 [file behavsci-15-00625-s001.zip › behavsci-3534857-supplementary.pdf]

Table S1. Impact of Parenting Avoidance (IPA)

Scoring Instructions: Sum all items to generate a total score for parenting inflexibility.

Please think of your role as a parent and respond to each statement by marking one box per row.

	Never	Rarely	Sometimes	Often	Always
My painful thoughts and feelings get in the way of how I want to parent.	1	2	3	4	5
My emotions cause problems in my relationship with my child.	1	2	3	4	5
I dwell on my parenting and what I would do differently next time.	1	2	3	4	5
It's hard to make parenting decisions because I'm afraid of making a mistake.	1	2	3	4	5
My worries about my child get in the way of successful parenting.	1	2	3	4	5
I worry about how other people might judge my parenting.	1	2	3	4	5
Before I allow my child to do something, I must have all my fears worked out.	1	2	3	4	5

Supplementary Tables S2–S9

Table S2

Regression summary for incremental validity of the Impact of Parenting Avoidance (IPA) scale

	Model 1				Model 2				Model 2 Summary		
	B	**β**	***t***	***p***	***B***	**β**	***t***	***p***	R^2	ΔR^2	ΔF
**Negative Parenting**											
AAQ-II	1.30	0.84	14.75	<0.001	0.63	0.41	3.00	0.004			
IPA					1.16	0.48	3.51	<0.001	0.75	0.04	12.35
**Stress**											
AAQ-II	0.46	0.87	16.62	<0.001	0.43	0.80	6.59	<0.001			
IPA					0.06	0.08	0.62	0.539	0.76	0.00	0.38
**Anxiety**											
AAQ-II	0.50	0.87	16.62	<0.001	0.41	0.70	5.67	<0.001			
IPA					0.17	0.19	1.49	0.140	0.77	0.01	2.22
**Depression**											
AAQ-II	0.52	0.87	16.30	<0.001	0.41	0.69	5.19	<0.001			
IPA					0.18	0.19	1.44	0.153	0.76	0.01	2.08
**Negative Parenting**											
AAQ-II	1.13	0.73	5.58	<0.001	0.56	0.36	2.11	0.038			
DASS	0.11	0.12	0.89	0.377	0.05	0.05	0.42	0.678			
IPA					1.13	0.47	3.15	0.002	0.74	0.03	9.92

Note. AAQ-II = acceptance and action questionnaire, second edition. DASS = depression, anxiety, and stress scales.

Table S3

Regression summary for incremental validity of the Parenting Specific Psychological Flexibility (PSPF) scale

	Model 1				Model 2				Model 2 Summary		
	B	β	*t*	*p*	*B*	β	*t*	*p*	R^2	ΔR^2	ΔF
**Negative Parenting**											
AAQ-II	1.32	0.85	15.20	<0.001	0.63	0.40	2.48	0.015			
PSPF					0.80	0.47	2.90	0.005	0.74	0.02	8.40
**Stress**											
AAQ-II	0.46	0.87	16.67	<0.001	0.34	0.63	4.11	<0.001			
PSPF					0.15	0.25	1.66	0.100	0.76	0.01	2.76
**Anxiety**											
AAQ-II	0.50	0.87	16.73	<0.001	0.25	0.43	2.93	0.004			
PSPF					0.29	0.47	3.17	0.002	0.78	0.03	10.05
**Depression**											
AAQ-II	0.52	0.87	16.70	<0.001	0.17	0.28	1.99	0.050			
PSPF					0.40	0.62	4.40	<0.001	0.79	0.04	19.38
**Negative Parenting**											
AAQ-II	1.12	0.72	5.57	<0.001	0.62	0.40	2.21	0.030			
DASS	0.13	0.14	1.06	0.293	0.03	0.03	0.22	0.825			
PSPF					0.76	0.45	2.54	0.013	0.73	0.02	6.46

Note. AAQ-II = acceptance and action questionnaire, second edition. DASS = depression, anxiety, and stress scales.

Table S4

Regression summary for incremental validity of the Parenting Acceptance and Action Questionnaire (PAAQ)

	**Model 1**				**Model 2**				**Model 2 Summary**		
	B	**β**	***t***	***p***	***B***	**β**	***t***	***p***	R^2	ΔR^2	ΔF
**Negative Parenting**											
AAQ-II	1.31	0.85	14.92	<0.001	1.02	0.66	7.40	<0.001			
PAAQ					0.43	0.24	2.65	0.010	0.74	0.02	7.02
**Positive Parenting**											
AAQ-II	-0.09	-0.12	-1.10	0.274	0.02	0.03	0.17	0.870			
PAAQ					-0.17	-0.19	-1.05	0.296	0.03	0.01	1.11
**Stress**											
AAQ-II	0.47	0.88	17.49	<0.001	0.43	0.82	9.84	<0.001			
PAAQ					0.05	0.08	1.00	0.320	0.78	0.00	1.00
**Anxiety**											
AAQ-II	0.50	0.89	18.46	<0.001	0.49	0.86	10.83	<0.001			
PAAQ					0.03	0.05	0.60	0.547	0.80	0.00	0.37
**Depression**											
AAQ-II	0.53	0.88	17.47	<0.001	0.47	0.78	9.60	<0.001			
PAAQ					0.09	0.13	1.54	0.126	0.78	0.01	2.38
**Negative Parenting**											
AAQ-II	0.97	0.63	4.41	<0.001	0.76	0.50	3.34	0.001			
DASS	0.22	0.24	1.68	0.098	0.18	0.19	1.36	0.176			
PAAQ					0.40	0.22	2.45	0.017	0.74	0.02	5.99
**Positive Parenting**											
AAQ-II	0.22	0.29	1.07	0.289	0.27	0.36	1.25	0.215			
DASS	-0.19	-0.41	-1.53	0.129	-0.17	-0.37	-1.35	0.182			
PAAQ					-0.13	-0.14	-0.76	0.448	0.04	0.01	0.58

Note. AAQ-II = acceptance and action questionnaire, second edition. DASS = depression, anxiety, and stress scales.

Table S5
Regression summary for incremental validity of the Parental Action Questionnaire (6-PAQ)

	**Model 1**				**Model 2**				**Model 2 Summary**		
	B	**β**	***t***	***p***	***B***	**β**	***t***	***p***	R^2	ΔR^2	ΔF
**Negative Parenting**											
AAQ-II	1.34	0.84	14.41	<0.001	0.67	0.42	4.90	<0.001			
6-PAQ					1.02	0.51	5.91	<0.001	0.80	0.09	34.94
**Positive Parenting**											
AAQ-II	-0.06	-0.08	-0.70	0.487	0.46	0.62	3.71	<0.001			
6-PAQ					-0.81	-0.84	-5.07	<0.001	0.24	0.23	25.72
**Stress**											
AAQ-II	0.47	0.87	16.11	<0.001	0.43	0.80	8.76	<0.001			
6-PAQ					0.06	0.09	0.97	0.337	0.75	0.00	0.93
**Anxiety**											
AAQ-II	0.49	0.86	15.51	<0.001	0.36	0.63	7.16	<0.001			
6-PAQ					0.21	0.29	3.29	0.001	0.77	0.03	10.85
**Depression**											
AAQ-II	0.52	0.86	15.63	<0.001	0.38	0.62	6.88	<0.001			
6-PAQ					0.22	0.29	3.20	0.002	0.77	0.03	10.25
**Negative Parenting**											
AAQ-II	1.13	0.71	5.36	<0.001	0.72	0.45	3.71	<0.001			
DASS	0.13	0.14	1.05	0.297	-0.04	-0.04	-0.36	0.724			
6-PAQ					1.03	0.52	5.68	<0.001	0.79	0.09	32.25
**Positive Parenting**											
AAQ-II	0.21	0.28	1.13	0.260	0.472	0.61	2.74	0.008			
DASS	-0.17	-0.36	-1.49	0.140	0.05	0.11	0.46	0.647			
6-PAQ					-0.90	-0.92	-4.92	<0.001	0.26	0.23	24.16

Note. AAQ-II = acceptance and action questionnaire, second edition. DASS = depression, anxiety, and stress scales.

Table S6
Regression summary for incremental validity of the Parental Psychological Flexibility questionnaire, Cognitive Defusion subscale (PPF-CD)

	Model 1				Model 2				Model 2 Summary		
	B	β	*t*	*p*	*B*	β	*t*	*p*	R^2	ΔR^2	ΔF
**Negative Parenting**											
AAQ-II	1.33	0.84	14.86	<0.001	0.24	0.15	1.13	0.261			
PPF-CD					-0.98	-0.74	-5.51	<0.001	0.79	0.07	30.31
**Stress**											
AAQ-II	0.47	0.87	16.63	<0.001	0.39	0.71	5.53	<0.001			
PPF-CD					-0.08	-0.17	-1.33	0.196	0.76	0.01	1.76
**Anxiety**											
AAQ-II	0.50	0.86	16.11	<0.001	0.29	0.50	3.97	<0.001			
PPF-CD					-0.20	-0.40	-3.14	0.002	0.77	0.03	9.84
**Depression**											
AAQ-II	0.52	0.86	16.09	<0.001	0.31	0.51	3.86	<0.001			
PPF-CD					-0.19	-0.38	-2.84	0.006	0.76	0.02	8.05
**Negative Parenting**											
AAQ-II	1.13	0.72	5.48	<0.001	0.25	0.16	1.02	0.313			
DASS	0.13	0.14	1.07	0.290	-0.00	-0.00	-0.01	0.988			
PPF-CD					-0.98	-0.73	-5.15	<0.001	0.78	0.07	26.57

Note. AAQ-II = acceptance and action questionnaire, second edition. DASS = depression, anxiety, and stress scales.

Table S7

Regression summary for incremental validity of the Psychological Flexibility questionnaire, Committed Action subscale (PPF-CA)

	Model 1				Model 2				Model 2 Summary		
	B	β	*t*	*p*	*B*	β	*t*	*p*	R^2	ΔR^2	ΔF
**Negative Parenting**											
AAQ-II	1.32	0.85	15.20	<0.001	0.78	0.50	4.10	<0.001			
PPF-CA					-0.91	-0.38	-3.13	0.002	0.74	0.03	9.78
**Stress**											
AAQ-II	0.46	0.87	16.66	<0.001	0.36	0.68	6.00	<0.001			
PPF-CA					-0.18	-0.21	-1.88	0.064	0.76	0.01	3.52
**Anxiety**											
AAQ-II	0.50	0.87	16.73	<0.001	0.43	0.75	6.50	<0.001			
PPF-CA					-0.12	-0.14	-1.21	0.229	0.76	0.00	1.47
**Depression**											
AAQ-II	0.52	0.87	16.67	<0.001	0.50	0.84	7.23	<0.001			
PPF-CA					-0.03	-0.03	-0.28	0.778	0.75	0.00	0.08
**Negative Parenting**											
AAQ-II	1.12	0.72	5.57	<0.001	0.62	0.40	2.46	0.016			
DASS	0.13	0.14	1.06	0.293	0.10	0.11	0.89	0.374			
PPF-CA					-0.92	-0.39	-3.09	0.003	0.74	0.03	9.54

Note. AAQ-II = acceptance and action questionnaire, second edition. DASS = depression, anxiety, and stress scales.

Table S8

Regression summary for incremental validity of the Psychological Flexibility questionnaire, Acceptance subscale (PPF-AC)

	Model 1				Model 2				Model 2 Summary		
	B	**β**	***t***	***p***	***B***	**β**	***t***	***p***	R^2	ΔR^2	ΔF
**Positive Parenting**											
AAQ-II	-0.07	-0.10	-0.92	0.363	-0.12	-0.16	-2.00	0.049			
PPF-AC					0.96	0.64	7.85	<0.001	0.42	0.41	61.54
**Positive Parenting**											
AAQ-II	0.22	0.28	1.18	0.243	0.04	0.05	0.30	0.765			
DASS	-0.19	-0.39	-1.64	0.105	-0.09	-0.19	-1.08	0.282			
PPF-AC					1.12	0.70	8.93	<0.001	0.51	0.48	79.68

Note. AAQ-II = acceptance and action questionnaire, second edition. DASS = depression, anxiety, and stress scales.

Table S9

Multiple regression summary for measures that demonstrated incremental validity above general flexibility and distress

	B	β	*t*	*p*	R^2
**Negative Parenting**					0.83
IPA	0.41	0.17	1.26	0.212	
PPF-CA	-0.51	0-.21	-1.80	0.075	
PPF-CD	-0.35	-0.27	-1.27	0.210	
PSPF	-0.04	-0.02	-0.12	0.903	
6-PAQ	0.69	0.36	3.55	<0.001	
PAAQ	-0.03	-0.02	-0.20	0.844	
**Negative Parenting - Adjusted**					0.82
IPA	0.58	0.24	2.08	0.041	
PPF-CA	-0.70	-0.29	-2.85	0.006	
6-PAQ	0.82	0.43	4.80	<0.001	
PAAQ	0.01	0.01	0.09	0.929	
**Positive Parenting**					0.48
PPF-AC	0.92	0.60	7.40	<0.001	
6-PAQ	-0.21	-0.21	-2.57	0.012	
**Anxiety**					0.76
PSPF	0.30	0.48	2.78	0.007	
6-PAQ	0.11	0.15	1.40	0.166	
PPF-CD	-0.13	-0.28	-1.51	0.136	
**Depression**					0.78
PSPF	0.47	0.71	4.21	<0.001	
6-PAQ	0.11	0.15	1.48	0.142	
PPF-CD	-0.02	0-.04	-0.22	0.827	

Note. IPA = impact of parenting avoidance. PPF-CA = parental psychological flexibility – committed action. PPF-CD = parental psychological flexibility – cognitive defusion. PSPF = parenting specific psychological flexibility. 6-PAQ = parental acceptance questionnaire. PAAQ = parental acceptance and action questionnaire. PPF-AC = parental psychological flexibility – acceptance.
